# Modulation of endothelin receptors in the failing right ventricle of the heart and vasculature of the lung in human pulmonary arterial hypertension

**DOI:** 10.1016/j.lfs.2014.02.020

**Published:** 2014-11-24

**Authors:** Rhoda E. Kuc, Myrna Carlebur, Janet J. Maguire, Peiran Yang, Lu Long, Mark Toshner, Nicholas W. Morrell, Anthony P. Davenport

**Affiliations:** a Clinical Pharmacology Unit, Addenbrooke's Hospital, Cambridge CB2 0QQ, UK; b Department of Medicine, Addenbrooke's Hospital, Cambridge CB2 0QQ, UK

**Keywords:** Pulmonary arterial hypertension, Endothelin, FR139317, Radioligand binding, Affinity constant, Receptor density

## Abstract

**Aims:**

In pulmonary arterial hypertension (PAH), increases in endothelin-1 (ET-1) contribute to elevated pulmonary vascular resistance which ultimately causes death by right ventricular (RV) heart failure. ET antagonists are effective in treating PAH but lack efficacy in treating left ventricular (LV) heart failure, where ET_A_ receptors are significantly increased. The aim was to quantify the density of ET_A_ and ET_B_ receptors in cardiopulmonary tissue from PAH patients and the monocrotaline (MCT) rat, which recapitulates some of the pathophysiological features, including increased RV pressure.

**Main methods:**

Radioligand binding assays were used to quantify affinity, density and ratio of ET receptors.

**Key findings:**

In RV from human PAH hearts, there was a significant increase in the ratio of ET_A_ to ET_B_ receptors compared with normal hearts. In the RV of the MCT rat, the ratio also changed but was reversed. In both human and rat, there was no change in LV. In human PAH lungs, ET_A_ receptors were significantly increased in the medial layer of small pulmonary arteries with no change detectable in MCT rat vessels.

**Significance:**

Current treatments for PAH focus mainly on pulmonary vasodilatation. The increase in ET_A_ receptors in arteries provides a mechanism for the beneficial vasodilator actions of ET antagonists. The increase in the ratio of ET_A_ in RV also implicates changes to ET signalling although it is unclear if ET antagonism is beneficial but the results emphasise the unexploited potential for therapies that target the RV, to improve survival in patients with PAH.

## Graphical abstract

**Figure:**
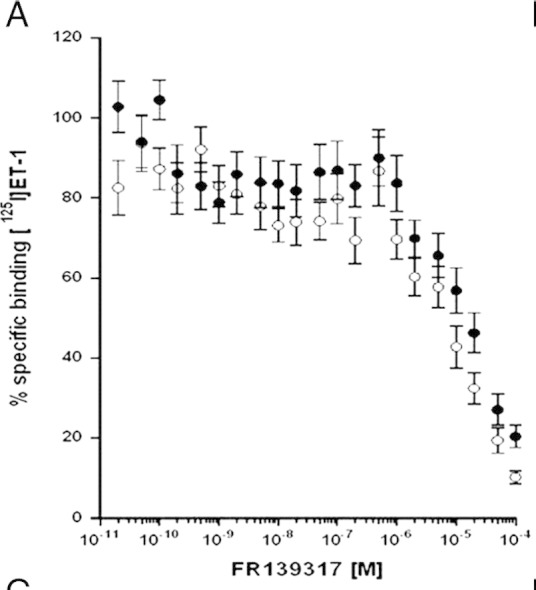

## Introduction

In pulmonary arterial hypertension (PAH), increases in endothelin-1 (ET-1) contribute to elevated pulmonary vascular resistance which ultimately causes death by right ventricular heart failure. PAH involves injury to the pulmonary vasculature producing elevations in pulmonary arterial pressure. As PAH progresses, chronic pressure and volume overload cause alteration of the structure of the right ventricle (RV) including hypertrophy and dilatation. As a result, the space taken up by the RV in the pericardium increases, impeding left ventricular (LV) diastolic filling, reducing LV end-diastolic volume and altering the LV contractile function (Bogaard et al., 2009). Right heart failure is the major cause of death in PAH patients. ET antagonists are effective in treating PAH (Liu et al., 2013) but in marked contrast, lack efficacy in treating left ventricular heart failure (Kelland and Webb, 2007; Kohan et al., 2012). This is surprising as the density of ET_A_ receptors in the LV of patients with ischaemic heart disease is significantly increased by 50%, compared with non-failing hearts (Peter and Davenport, 1996a). However measurement of receptor density in the RV from patients with PAH using radioligand binding assays has not been studied.

ET_A_ receptors are the principal sub-type in the medial or smooth muscle layer of human blood vessels, including large epicardial and small resistance coronary arteries within the heart where ET_A_ receptors mediate vasoconstriction (Maguire and Davenport, 1995; Pierre and Davenport, 1998). We have previously shown that in human large conduit vessels these are altered in cardiovascular disease including PAH (Kuc and Davenport, 2000). Davie et al. (2002) found no change in ratio of ET_A_:ET_B_ but increased overall receptor density in smooth muscle cells from human pulmonary small resistance arteries in PAH.

ET_B_ receptors localise to the endothelium and cause beneficial vasodilatation by the release of endothelium derived relaxing factors, opposing constrictor tone. In addition, in organs such as the lungs that are rich in ET_B_ receptors (Bagnall et al., 2006), this sub-type functions to clear ET-1 from the plasma (Johnström et al., 2005). Two classes of ET antagonist are used clinically, mixed antagonists that block both sub-types and ET_A_ selective drugs. The precise molecular mechanism whereby these antagonists produce benefit in PAH is not established. In particular, the contribution of ET_B_ receptors to the development of this condition and the need to block this sub-type as well as the ET_A_ is still unclear (Vachiery and Davenport, 2009). Our aim was to compare the density of both ET receptor sub-types in surgical samples from the right and LV of hearts and lungs removed from PAH patients at the time of transplantation, in comparison with normal tissues. Secondly to measure receptor density in a widely use animal model of PAH, which recapitulates a majority of the features of the human condition including right ventricular failure (Ryan et al., 2011).

## Materials and methods

### Human heart

Surgical samples of LV and RV were obtained from PAH patients (idiopathic pulmonary artery hypertension) undergoing heart–lung transplantation and from normal controls that were not suitable for transplantation. Samples of PAH lung were obtained from patients undergoing lung transplantation and histologically normal control tissue was from patients undergoing lung lobectomy procedures. All tissues were collected with informed consent and ethical approval.

### MCT-rat tissue collection

The procedures used in this study were approved by the local animal ethical committee and were performed under UK Home Office Project Licence authority; the study conformed to the National Institutes of Health Guidelines for the Care and Use of Laboratory Animals. Male Sprague–Dawley rats (approximately 250 g) received a single subcutaneous injection of monocrotaline (60 mg/kg) at day 0 to induce PAH (Long et al., 2013). The rats were maintained for three weeks following injection to develop muscularization of small pulmonary arteries in the lungs and right ventricular hypertrophy but without developing dilated heart failure (Long et al., 2013). Rats were euthanized by CO_2_ inhalation. Organs were removed and snap-frozen in liquid nitrogen and stored at − 70 °C until further use.

### Competition assays

Cryostat-cut tissue sections (10 μm) were mounted onto gelatine coated microscope slides.

Competition binding assays were performed as previously described (Maguire et al., 2012a), to determine the affinities (K_D_) and maximum densities (B_MAX_) of ET_A_ and ET_B_ receptors.

Sections were incubated with 0.1 nM [^125^I]-ET-1 (Perkin Elmer) and increasing concentrations (20 pM–10 μM) of the ET_A_ selective agonist FR139317 for 2 h at 23 °C. Non-specific binding (NSB) was determined using 1 μM of unlabelled ET-1. Following incubation and washing (3 × 5 min) in ice-cold Tris–HCl buffer to break the equilibrium, sections were counted in a gamma counter.

Competition curves were obtained by plotting specific binding as a percentage of total binding (binding in the absence of competitor) against the log concentration of the competing ligand. The data were analysed (see Maguire et al., 2012b) using non-linear iterative curve fitting programmes (KELL, containing EBDA and LIGAND programmes, Biosoft, Cambridge UK) to calculate K_D_ (affinity constant) and B_MAX_ (maximum density of receptors).

### Autoradiography

For autoradiographical analysis, binding was carried as previously described (Ling et al., 2012) using assay conditions outlined above in a set of adjacent sections, to determine total [^125^I]-ET-1 (0.1 nM) binding, non-specific binding (1 μM unlabelled ET-1) and with selective antagonists, either 0.1 μM BQ3020 or 0.1 μM FR139317 to determine ET_A_ and ET_B_ receptor distribution respectively. Adjacent sections were stained to facilitate histological identification of pulmonary vasculature. Sections were washed to break the equilibrium and apposed, together with calibrated radioactive standards, to radiation-sensitive film (Kodak BioMax MR-1, Perkin Elmer). Resulting autoradiograms were analysed by measuring diffuse integrated optical density using the Quantimet 970 image analysis system. ET-1 receptor density was measured by digitizing each autoradiographical image and regions of interest on tissue sections were delineated. Optical densities were converted to specifically bound radioligand by interpolation from standard curves and subtraction of non-specific binding in an adjacent section.

## Results

### Pharmacodynamic parameters in human and rat heart

**Fig. 1:**
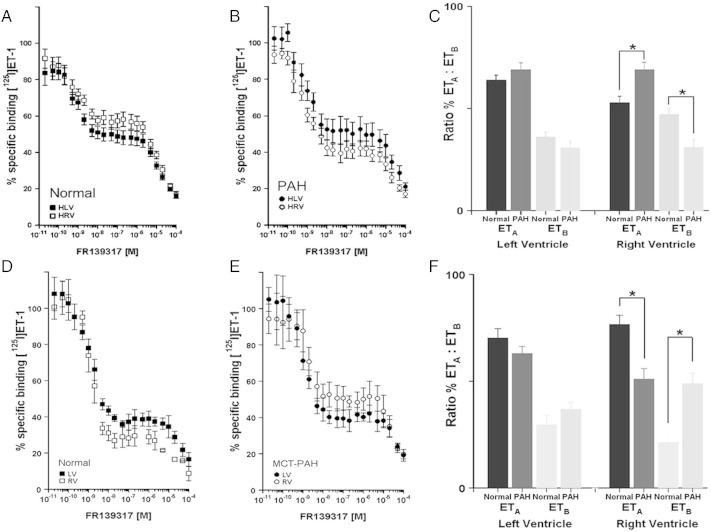
A–C Competition of FR139317 for [^125^I]-ET-1 binding in human left (LV) and right (RV) ventricles from (A) normal controls and (B) patients transplanted for PAH. A biphasic curve was obtained as expected in each case corresponding to a high affinity ET_A_ and low affinity ET_B_ site, allowing the affinities (K_D_), densities (B_MAX_) and ratio of the two sub-types to be measured. (C) Comparison of ET sub-type ratio in LV and RV in hearts from normal controls and patients with PAH showing a significant increase in ET_A_ but decrease in ET_B_ in the right ventricle (n = 12 PAH and 9 control individuals, mean ± s.e.mean; Student's t-test, *p < 0.05 t-test).

In human normal hearts, competition binding revealed the expected ratio of ET_A_ to ET_B_ receptors (Fig. 1A, Peter and Davenport, 1996a). FR139317 competed biphasically for the binding of [^125^I]-ET-1, with a two-site fit preferred over a one-site model with no significant difference in affinity constants (Table 1, K_D_) between patient groups (Fig. 1). Whilst there was no significant change in receptor sub-type ratio in LV, there is a significant increase in ET_A_ with a concomitant decrease in ET_B_ receptors in the failing RV (Fig. 1B, C).

In the rat model (Fig. 1D) the expected ratio of receptor sub-types was observed in both chambers of the hearts of control rats (Peter and Davenport, 1996b). In the MCT rat, receptor density was significantly different in the RV compared with vehicle control but with ET_A_ downregulation and ET_B_ upregulation (Fig. 1E, F). These changes led to a significant shift in relative ET_A_:ET_B_ receptor density ratio from 73:27 in control rat RV to 51:49 in MCT-rat RV. In the LV, no significant difference in ET_A_ and ET_B_ receptor density in MCT-rat heart compared to controls was observed (Table 2).

### Pharmacodynamic parameters in human PAH and MCT lung

**Fig. 2:**
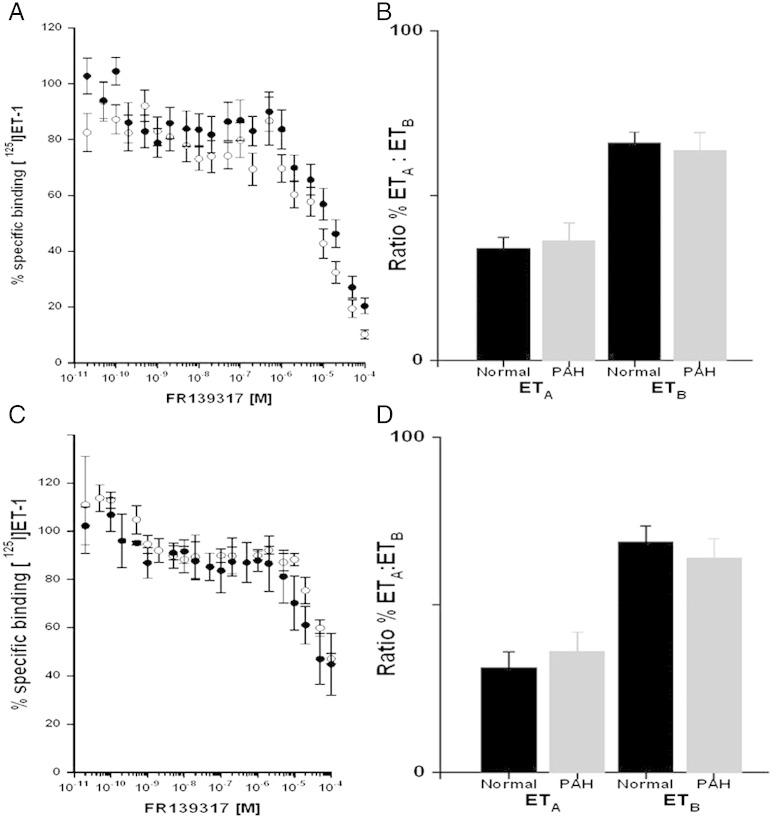
Competition of FR139317 for [^125^I]-ET-1 binding to human lung from (A) normal controls and patients transplanted for PAH. A biphasic curve was obtained as expected in each case corresponding to a high affinity ET_A_ and low affinity ET_B_ site (B) Comparison of ET sub-type ratio in the lungs from normal controls and patients with PAH showing no significant change in the ratio of either sub-type (n = 8 PAH and 7 control individuals, mean ± s.e.mean). (C) Competition of FR139317 for [^125^I]-ET-1 binding in rat lung controls and MCT rats. A biphasic curve was also obtained as expected in each case corresponding to a high affinity ET_A_ and low affinity ET_B_ site. (D) Comparison of ET sub-type ratio in the lungs of control and MCT treated rats, control and patients MCT arts with PAH showing no significant change in the ratio of either sub-type (n = 5 MCT and 3 control rats ± s.e.mean).

**Fig. 3:**
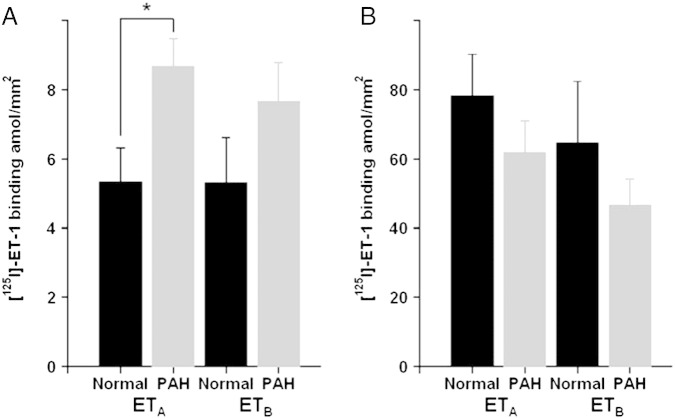
Autoradiographical analysis of the density (amol/mm^2^) in the binding of [^125^I]-ET-1 to ET_A_ and ET_B_ to the smooth muscle (medial) of small pulmonary arteries from PAH compared to normal control lung tissues showing. There was a significant increase in ET_A_ compared with normal vessels (A) but no change in ET_B_ (n = 8 PAH and 7 control individuals ± s.e.mean; *p < 0.05 t-test). In the lungs of MCT rats (B), there was no change (n = 6 MCT and 6 control rats ± s.e.mean).

Competition studies using whole cryostat sections in the lungs from patients with PAH compared to normal control tissues did not detect a significant difference in binding affinities (K_D_) for ET_A_ or ET_B_ and no change in receptor densities (B_MAX_) or ratio of sub-types in human PAH lungs compared with control (Fig. 2B, Table 3). In agreement, there were no changes in these parameters in MCT lungs compared with control. However, following apposition of labelled sections to radiation sensitive film, image analysis permitted the measurement of densities in discrete cell types. In the medial layer of small pulmonary arteries identified by comparison with adjacent stained sections, there was a significant increase in vascular ET_A_ receptors in PAH compared with control small vessels (Fig. 3A, Table 3). No equivalent changes were detected in the medial layer of MCT rat lungs compared with control (Fig. 3B, Table 3).

## Discussion

### Human heart with PAH

We have previously shown that ET_A_ receptors in the failing LV of patients with ischaemic heart disease are significantly increased by 50% (Peter and Davenport, 1996a,b). In agreement, in the failing RV of patients with PAH, there was a significant increase in the ratio of ET_A_ receptors in ET_B_ density. In agreement, Nagendran et al. (2013) using semi-quantitative immunocytochemistry also found an increase in ET_A_ expression in RV of patients with PAH. It is well established that levels of ET-1 are higher in PAH patients (Stewart et al., 1991). In addition, in animal models, clearance and internalization of ET-1 by ET_B_ receptors are critical in preventing circulating ET-1 from binding to ET_A_ receptors in the heart (Johnström et al., 2005) and the reduction that we observed in the RV in ET_B_ could contribute further to tissue levels. Taken together, these results suggest the potential for increased inotropic action via the ET_A_ sub-type. The RV of PAH patients is subjected to both pressure overload and autocrine/paracrine mediators such as ET-1, whereas the LV is only subjected to the latter. The RV and LV also have different embryological origins (Farha et al., 2013) and may respond differently to stressors and to therapies.

In PAH, the main benefit of ET antagonists may block deleterious vascular effects rather than improve cardiac function. In support of this hypothesis, in this study we have been able to determine, using autoradiography, the ET receptor densities within the smaller vessels of the tertiary structures of the lung demonstrating, in agreement with Davie et al. 2002, a ratio 50:50 for ET_A_ to ET_B_ receptors with a significant increase in the ET_A_ subtype in PAH vessels compared to control lung vessels. There was no change in ET_B_ receptors, consistent with results in other human diseased vessels (Maguire and Davenport, 2000).

Modulation of ET receptors in the RV of PAH patients suggests an adaptive response to both the pressure overload and changes in autocrine/paracrine mediators, such as ET-1, experienced by these patients. In heart failure, increased receptor density may be an adaptive response to increase beneficial cardiac contractility. ET receptor antagonists may therefore decrease RV function. In a clinical trial comparing bosentan, a mixed ET antagonist with sidenafil, both decreased pulmonary arterial pressure to the same extent but unlike sildenafil, bosentan failed to improve RV ejection (Wilkins et al., 2005).

### Rat model of PAH

In agreement with human PAH, there was no change in ET receptor density in the LV but ET receptor density was changed in the RV in the MCT rat, albeit the ratio was reversed compared with human. In addition, we did not detect an equivalent increase in receptor density in the medial layer of small arteries in MCT rat lungs. This may be because although MCT-treated rats develop PAH with marked RV hypertrophy, MCT causes these changes within three weeks, whereas patients undergoing transplantation of heart and lungs are in the later stages of the disease. There is a consensus in the rat model of pulmonary hypertension that neither RV systolic pressure nor function is improved in this model by the mixed receptor antagonist, bosentan (see for example, Hill et al., 1997; Jiang et al., 2011). Interestingly, selective ET_A_ antagonists have demonstrated efficacy in this model (Schroll et al., 2008; Kosanovic et al., 2011).

## Conclusion

The main finding in this study is that there was a significant increase in the ratio of ET_A_ to ET_B_ in the RV from human PAH hearts compared with control, implying that this could translate into an increase in inotropic action by ET, particularly as levels of the peptide also elevated in PAH. Currently, ET_A_ and mixed ET_A_/ET_B_ receptor antagonists are both effective in the treatment of PAH. Both would be expected to block any increased inotropic action mediated by ET, suggesting that the inotropic effect is not a major benefit in the RV of PAH patients where the RV is characterised by hypertrophy. ET_A_ receptors also increase in the LV of patients with heart failure but in marked contrast to PAH, ET receptor antagonists have not fulfilled their expected promise in clinical trials; beneficial positive inotropic actions of ET may be more important in cardiomyopathy. Thus whilst ET antagonists in left ventricular heart failure produce the desired vasodilatation, this is offset by fluid retention and activation of the renin hypertension system. In PAH, ET antagonists effectively cause the desired pulmonary vasodilatation whereas blocking cardiac ET receptors has little impact on clinical worsening.

PAH is characterized by high pulmonary vascular resistance and vascular remodelling, which results in RV afterload and subsequent failure. Current treatments for PAH including ET antagonists have tended to focus on pulmonary vasodilatation. This study has confirmed the upregulation of ET_A_ receptors in the pulmonary vasculature of PAH patients where the known vasodilator properties of ET antagonists are of benefit. Crucially, the study has also provided evidence for an increase in the ratio of ET_A_ receptors in the RV, implying changes to the ET signalling pathway but it is unclear whether ET antagonism is beneficial. These results emphasise the still unexploited potential for therapies that target the RV, with the aim of supporting the RV of the heart, to improve survival in patients with PAH (Sitbon and Morrell, 2012).

## Conflict of interest

The authors declare that there are no conflicts of interest.

## Figures and Tables

**Table 1:** K_D_ (affinity constant) and B_MAX_ (maximum density of receptors) values for ET_A_ and ET_B_ receptor subtypes human left and right ventricles from normal controls and patients transplanted for PAH.

Human heart		ET_A_ K_D_ (nM)	ET_A_ B_MAX_ (fmol/mg)	ET_B_ K_D_ (μM)	ET_B_ B_MAX_ (fmol/mg)	Ratio % ET_A_:ET_B_
Normal (n = 12)	LV	0.60 ± 0.01	66.1 ± 4.2	44.9 ± 3.2	44.6 ± 8.5	64:36
	RV	0.41 ± 0.11	69.2 ± 5.1	28.7 ± 1.9	75.7 ± 18.1	52:48
PAH (n = 9)	LV	0.76 ± 0.25	85.3 ± 11.4	36.2 ± 4.9	38.3 ± 6.4	69:31
	RV	0.45 ± 0.08	80.8 ± 7.8	36.5 ± 2.9	37.0 ± 5.5	⁎69:⁎31

n = 12/9 individuals, mean ± s.e.mean.

⁎ p < 0.05 t-test.

**Table 2:** Comparison of ET sub-type ratio in LV and RV in hearts from normal control rats and MCT rats with PAH.

Rat heart		ET_A_ K_D_ (nM)	ET_A_ B_MAX_ (fmol/mg)	ET_B_ K_D_ (μM)	ET_B_ B_MAX_ (fmol/mg)	Ratio % ET_A_:ET_B_
Control (n = 6)	LV	1.10 ± 0.29	194.0 ± 24.9	49.4 ± 8.99	80.3 ± 13.2	70.3:29.7
	RV	0.77 ± 0.18	346.7 ± 42.8	28.7 ± 7.53	89.0 ± 16.3	73.4:26.6
MCT (n = 6)	LV	0.66 ± 0.11	167.9 ± 22.7	56.7 ± 2.02	94.1 ± 9.30	63.1:36.9
	RV	0.79 ± 0.07	147.4 ± 20.0	47.3 ± 7.85	139.7 ± 14.8	⁎51.1:⁎48.9

n = 5 individuals ± s.e.mean.

⁎ p < 0.05 t-test.

**Table 3:** K_D_ (affinity constant) and B_MAX_ (maximum density of receptors) values for ET_A_ and ET_B_ receptor subtypes in human lung from normal controls and patients transplanted for PAH (n = 12 and 8 individuals) and rat lungs from control and monocrotaline treated animals (n = 3 and 5 animals), data are mean ± s.e.mean, No significant difference from control values (Student's t-test) at p < 0.05 was detected.

		ET_A_ K_D_ (nM)	ET_A_ B_MAX_ (fmol/mg)	ET_B_ K_D_ (μM)	ET_B_ B_MAX_ (fmol/mg)	Ratio % ET_A_:ET_B_
Human	Normal (n = 7)	2.05 ± 1.09	128.5 ± 19.0	13.4 ± 0.81	257.5 ± 48.0	34:66
	PAH (n = 8)	1.67 ± 2.49	137.0 ± 38.6	13.8 ± 1.11	181.0 ± 30.3	36:64
Rat	Control (n = 3)	5.24 ± 7.40	63.4 ± 25.8	55.9 ± 9.45	125.8 ± 34.6	31:69
	MCT (n = 5)	0.58 ± 0.45	134.6 ± 57.0	63.5 ± 4.89	210.1 ± 50.1	36:64
